# Identification of a rare de novo three-way complex t(5;20;8)(q31;p11.2;p21) with microdeletions on 5q31.2, 5q31.3, and 8p23.2 in a patient with hearing loss and global developmental delay: case report

**DOI:** 10.1186/1755-8166-2-2

**Published:** 2009-01-07

**Authors:** Roland Haj, Kelly Jackson, Beth A Torchia, Lisa G Shaffer, Bassem A Bejjani, Gordon C Gowans, Michael E Ruff

**Affiliations:** 1 Signature Genomic Laboratories, Spokane, WA, USA; 2 Weisskopf Child Evaluation Center, Department of Pediatrics, University of Louisville, Louisville, KY, USA; 3 Jasper Pediatric Associates, Jasper, IN, USA

## Abstract

**Background:**

Complex chromosome rearrangements (CCRs), which involve more than two breakpoints on two or more chromosomes, are uncommon occurrences. Although most CCRs appear balanced at the level of the light microscope, many demonstrate cryptic, submicroscopic imbalances at the translocation breakpoints.

**Results:**

We report a female with hearing loss and global developmental delay with a complex three-way unbalanced translocation (5;20;8)(q31;p11.2;p21) resulting in microdeletions on 5q31.2, 5q31.3, and 8p23.2 identified by karyotyping, microarray analysis and fluorescence in situ hybridization.

**Discussion:**

The microdeletion of bands 8p23.2 may be associated with the hearing impairment. Furthermore, the characterization of this patient's chromosomal abnormalities demonstrates the importance of integrated technologies within contemporary cytogenetics laboratories.

## Background

Reciprocal de novo translocations occur in about 1 in 2,000 newborns [1]. Although balanced translocations are not often associated with abnormal phenotypes, unbalanced translocations resulting in deleted or altered gene sequences usually cause appreciable clinical features [2,3]. Even more unusual than unbalanced translocations are complex chromosome rearrangements (CCRs). CCRs are structural abnormalities that involve more than two breakpoints and exchange of genetic material between two or more chromosomes [4,5]. The most common CCRs involve three chromosomes with breakpoints on each chromosome. The occurrence of three-way multiple translocations is rare and often difficult to distinguish from balanced translocations without the aid of additional diagnostic tools such as fluorescence in situ hybridization (FISH) [6] or microarray-based comparative genomic hybridization (array CGH) [7,8].

We report on an 8-month-old female with hearing loss, global developmental delay and myopathic face in whom we found a de novo complex translocation (5;20;8)(q31;p11.2;p21) and three microdeletions del(5)(q31.2q31.2), del(5)(q31.3q32) and del(8)(p23.2) identified by traditional and molecular cytogenetic methods.

## Case presentation

The proband is an 8-month-old female who was referred for global developmental delay and hearing loss. She was born at term by cesarean section to a 31-year-old mother. The proband's birth weight was 4 kg (90^th ^centile) and her length was 48 cm (25^th ^centile). The mother reports that she did not drink alcohol, smoke cigarettes, or use any unusual medications during the pregnancy. The father of the proband was 32 years old with no prior family history of congenital abnormalities.

**Figure 1:**
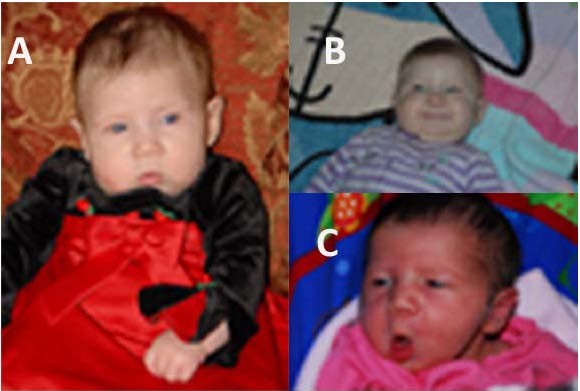
**Proband at 5 months of age (A,B) and 6 days of age (C)**. Note myopathic face.

At examination at 5 months of age, the patient was 6.6 kg (<50^th ^centile), 61 cm in length (<25^th ^centile), with a head circumference of 41.5 cm (50^th ^centile). An Auditory Brainstem Response test revealed bilateral mild sensorineural hearing loss. An MRI of the brain revealed white matter loss with thinning of the corpus callosum. Upon physical examination, the patient displayed minor dysmorphic features including a myopathic face (Fig 1). No other remarkable dysmorphic features or observable anomalies were noted.

## Results

**Figure 2:**
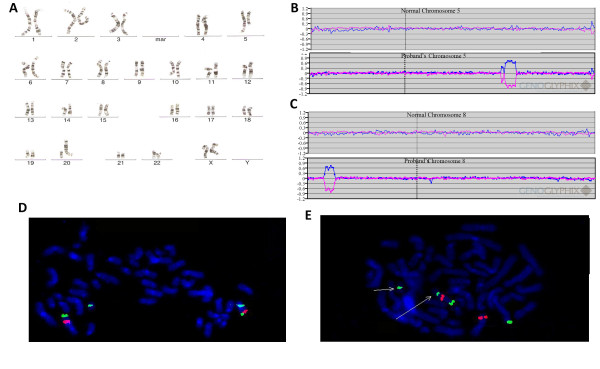
**Summary of characterization of t(5;20;8)(q31;p11.2;p21)**. (A) Karyotype results for the patient; note the abnormal chromosome 20. (B) Microarray results showing complex rearrangement of 5q. The top plot shows a normal chromosome 5; the bottom plot shows the abnormal chromosome 5. Each clone represented on the array is arranged along the x-axis according to its location on the chromosome with the most distal/telomeric p-arm clones on the left and the most distal/telomeric q-arm clones on the right. The blue line plots represent the ratios from the first experiment for the case (control Cy5/patient Cy3) and the pink plots represent the ratios obtained from the second experiment for the case in which the dyes have been reversed (patient Cy5/control Cy3). (C) Microarray results showing deletion of 8p23.2. Microarray plots are arranged as in B from pter to qter. (D) FISH results for 5q31.2 (red), 5q tel (green) and 20 cen (green). (E) Locations on the derivative chromosome of 8p22 (red), 5q tel (green), and 20 cen (green).

GTW-band analysis of the proband's peripheral blood showed a CCR with an apparent deletion on chromosome 5. The karyotype was determined to be 46,XX,t(5;20;8)(q31;p11;p21),del(5)(q31.3q33.1) (Fig. 2a). Microarray analysis demonstrated a single-copy loss of two regions on 5q: a two-BAC clone loss at 5q31.2 ~240 kb in size (CTD-2015J11 – RP11-264B21) and a nine-BAC clone loss at 5q31.3q32 (RP11-454M24 – RP11-436M5) of 4.1 Mb in size. The regions of copy-number loss were separated by 4 Mb of normal intervening sequence. Microarray analysis also demonstrated a single-copy loss of nine BACs at 8p23.2 (RP11-34M12 – CTD-2340K13) 1.5 Mb in size.

FISH was performed to confirm the results of the microarray analysis. A BAC clone from the deleted 5q31.2 region (RP11-264B21) showed a diminished signal on the short arm of the abnormal derivative chromosome 20 and no signal on the derivative 5, suggesting the BAC contains the breakpoint of the 5q31.2 deletion. The probe from the non-deleted region between the two 5q31 deletions (RP11-166J22) also hybridized to the der 20, as did a probe to the 8p22 region (RP11-447G11).

FISH using BAC clones from the deleted 5q31.3 region (RP11-1104L21) and the deleted 8p32.2 region (RP11-1148N20) confirmed the deletions at 5q31.3 and 8p23.2, respectively. The 5q telomeric probe (RP11-69N15) was used as a control, and it showed hybridization to the der 20. The 20p telomeric probe (RP11-530N10) was used as a second control, and it showed hybridization on the short arm of the abnormal derivative 8. The 8p telomeric probe (RP11-412M23) was used as a third control and hybridized to the long arm of the derivative 5.

Thus, this individual has a three-way complex translocation between the long arm of chromosome 5, the short arm of chromosome 20 and the short arm of chromosome 8 resulting in three interstitial deletions at bands 5q31.2, 5q31.3, and 8p23.2 with respective deletion sizes of approximately 240 kb, 4.1 Mb, and 1.5 Mb as estimated by array CGH.

## Discussion

We report the molecular and cytogenetic finding of a patient with a complex chromosome rearrangement involving 5q31, 20p11.2 and 8p21 resulting in microdeletions of bands 5q31.2, 5q31.3, and 8p23.2. The proband's phenotype is nondescript; global developmental delay and sensorineural hearing loss are associated with many microdeletions including 1p36, 7q21.13-q22.1, 11q25, and Xq21 [9-13]. Many genomic regions are associated with hearing loss because of the complexity of and number of genes involved in hearing development.

5q31.2, 5q31.3, and 8p23.2 may be potential regions of the genome critical to hearing development. However, abnormalities involving 5q are rarely associated with hearing loss, with the exception of Treacher Collins syndrome, an autosomally dominant trait associated with severe craniofacial malformation that maps to 5q32-5q33.1 [14]. Because the patient has no familial history of Treacher Collins or the characteristic facial features, this syndrome has been excluded as a possible diagnosis in this patient. The short arm of chromosome 8 may contain one or more genes directly or indirectly involved in the formation of functional hearing pathways. Masuda et al. [15] and Devriendt et al. [16] report sensorineural hearing loss in patients with 8p abnormalities proximal to the telomere resulting in partial 8p monosomy similar in size and location to the deletion in our patient. In contrast, Fryns et al. [17], Hutchinson et al. [18], and Devriendt et al. [19] report patients with deletions of 8p23.1pter without noting sensioneural hearing loss. CMSD1 is the only known gene in the 1.5 Mb region on 8p23.2 deleted in this individual. The deletion of CMSD1 has not been directly implicated in sensorineural hearing loss or a similar phenotype, but CMSD1 is known to play a role in neuronal migration and development of the central nervous system [20]. The deletion may be causing a position effect on the chromosome and may play a role in the proband's phenotype, either because the chromosomal rearrangement separated a promoter from its transcriptional regulatory element, resulting in gene silencing; the rearrangement juxtaposed a gene with an enhancer from another gene, leading to inappropriate gene expression; or the rearrangement moved a gene and its regulatory elements to a region of the genome that is transcriptionally silent, such as heterochromatin. Alternatively, the translocation breakpoints may have interrupted a gene or genes. The ascertainment of additional individuals with similar rearrangement breakpoints on 8p23.2 is necessary for further genotype-phenotype correlation.

Because of the complexity of the CCR, none of the technologies could accurately confirm the de novo complex CCR independently. Two of the microdeletions initially went undetected by traditional cytogenetic analysis, which is consistent with previous research on CCRs [7,8,21]. Only after identification of the approximate breakpoints by microarray analysis was it possible to determine the location and order of the de novo CCR within the patients' genome using the appropriate FISH probes to locate the translocated sections of chromosomes 8p and 5q. Our results suggest the successful integration of multiple cytogenetic techniques–karyotyping, aCGH, and FISH – is necessary in the diagnostic laboratory for the characterization of complex chromosomal rearrangements.

## Materials and methods

### Cytogenetic analysis

Cytogenetic analysis was performed on peripheral blood lymphocytes by G-banding according to standard procedures.

### BAC array CGH

Array CGH was performed with a bacterial artificial chromosome (BAC) microarray (the SignatureChip^®^; Signature Genomic Laboratories, Spokane, WA) that was developed for the detection of microdeletions, microduplications, aneuploidy, unbalanced translocations, and subtelomeric and pericentromeric copy-number alterations [22]. The current version of the SignatureChip, the SignatureChip Whole Genome™ (SignatureChipWG), contains 4670 BACs representing 1543 loci with each locus being represented by a minimum of three overlapping clones. The subtelomeric and pericentromeric regions are represented with a higher density of overlapping BAC clones, targeted to the unique sequences adjacent to these repetitive regions and consist of contigs of clones located approximately every 0.5 Mb spanning more than 5 Mb. Genes in important developmental pathways are also covered by contigs of BACs to fill in the chromosome arms and provide higher resolution with an average gap size between contigs of ~1.6 Mb [22].

Microarray analysis was performed as described [22], with the following modifications: Briefly, genomic DNA was extracted from peripheral blood using a Qiagen M48 Biorobot automated DNA extraction system. Purified genomic DNA was then sonicated and labeled with Alexaflour dyes 555 or 647 using a BioPrime Total DNA labeling kit (Invitrogen Corp). Microarrays were hybridized as previously described (8) and washed using a Little Dipper automated microarray washing station (SciGene). Microarrays were scanned on an Axon 4000B scanner (Molecular Devices) and signal intensity ratios were analyzed as described (8) using a custom analysis and display interface (Genoglyphix™).

### FISH

All abnormalities detected by array CGH were confirmed and visualized by metaphase or interphase fluorescence in situ hybridization (FISH) using one or more BAC clones determined to be abnormal by array CGH [23].

## Competing interests

R Haj and BA Torchia are employees of Signature Genomic Laboratories, LLC. LG Shaffer and BA Bejjani sit on the Members' Board of Signature Genomic Laboratories, LLC.

## Authors' contributions

RH wrote the manuscript; BAT signed out the molecular cytogenetic results; MWR referred the patient for study; KJ and GG contributed clinical information; LGS and BAB coordinated the study. All authors have read and approved the manuscript.

## Consent

This case report is presented with the consent of the patient's family.
